# RASSF10 suppresses hepatocellular carcinoma growth by activating P53 signaling and methylation of RASSF10 is a docetaxel resistant marker

**DOI:** 10.18632/genesandcancer.67

**Published:** 2015-05

**Authors:** Yongshuai Jin, Baoping Cao, Meiying Zhang, Qimin Zhan, James G. Herman, Miao Yu, Mingzhou Guo

**Affiliations:** ^1^ Department of Gastroenterology & Hepatology, Chinese PLA General Hospital, Beijing, China; ^2^ Department of Interventional Radiology, Chinese PLA General Hospital, Beijing, China; ^3^ Medical College of NanKai University, Tianjin, China; ^4^ State Key Laboratory of Molecular Oncology, Cancer Institute and Hospital, Chinese Academy of Medical Sciences & Peking Union Medical College, Beijing, China; ^5^ The Hillman Cancer Center, University of Pittsburgh Cancer Institute, Pittsburgh, PA, USA

**Keywords:** RASSF10, DNA methylation, P53 signaling, hepatocellular carcinoma, epigenetics

## Abstract

Hepatocellular carcinoma (HCC) is one of the most common malignances and the second leading cause of cancer related death worldwide. RASSF10 is located on chromosome 11p15.2, a region that shows frequent loss of heterozygosity (LOH) in several cancer types. Our previous study found that RASSF10 suppresses colorectal cancer growth by activating P53 signaling. To explore the epigenetic changes and the mechanism of RASSF10 in human HCC, 69 cases of primary HCC, twenty cases of normal liver tissue samples and 17 HCC cell lines were involved in this study. We found that RASSF10 was methylated in 82.6% (57/69) of human primary HCC and methylation of RASSF10 was significantly associated with tumor size (P < 0.05) and TNM stage (P < 0.05). The expression of RASSF10 was regulated by promoter region methylation. Restoration of RASSF10 expression suppressed cell proliferation, induced apoptosis and G2/M phase arrest, as well as sensitized cells to docetaxel and activated P53 signaling in HepG2 and QGY7703 cells. In conclusion, we demonstrated that RASSF10 is frequently methylated in human HCC and its methylation is a potential docetaxel resistant marker. Our data also indicate that RASSF10 suppresses human HCC growth by activating P53 signaling.

## INTRODUCTION

Hepatocellular carcinoma (HCC) is the fifth most common cancer in men and the ninth most common cancer in women, and it is the second leading cause of cancer related death worldwide [1]. The mechanisms involved in HCC remain unclear. Accumulations of genetic and epigenetic changes are regarded as important mechanisms for many cancers [2-5]. Aberrant epigenetic changes have been frequently found in human HCC [6-11]. The Ras-association domain family (RASSF) proteins are associated with Ras-like small GTP binding proteins and participate in a range of cellular processes including cell growth, adhesion, migration, differentiation and apoptosis [12, 13]. The family of genes comprises 10 members that are subdivided into C-terminal (RASSF1-6) and N-terminal (RASSF7–10) RASSF genes [14]. The C-terminal RASSF proteins harbor Ras association domains and Salvador/RASSF/Hippo (SARAH) domains in the C-terminus, whereas the N-terminal RASSF proteins contain Ras association domains in the N-terminus and lack the SARAH domain [15]. RASSF10 is located on chromosome 11p15.2 [16]. Loss of heterozygosity (LOH) frequently occurs in this region in several types of cancer [17-21]. RASSF10 is frequently methylated in many human cancers [13, 14, 22-26]. Our previous study found that RASSF10 suppresses colorectal cancer growth by activating P53 signaling. In this study, we explored the epigenetic changes and the role of RASSF10 in HCC progression.

## RESULTS

### RASSF10 is silenced by promoter region hypermethylation in HCC cells

**Figure 1:**
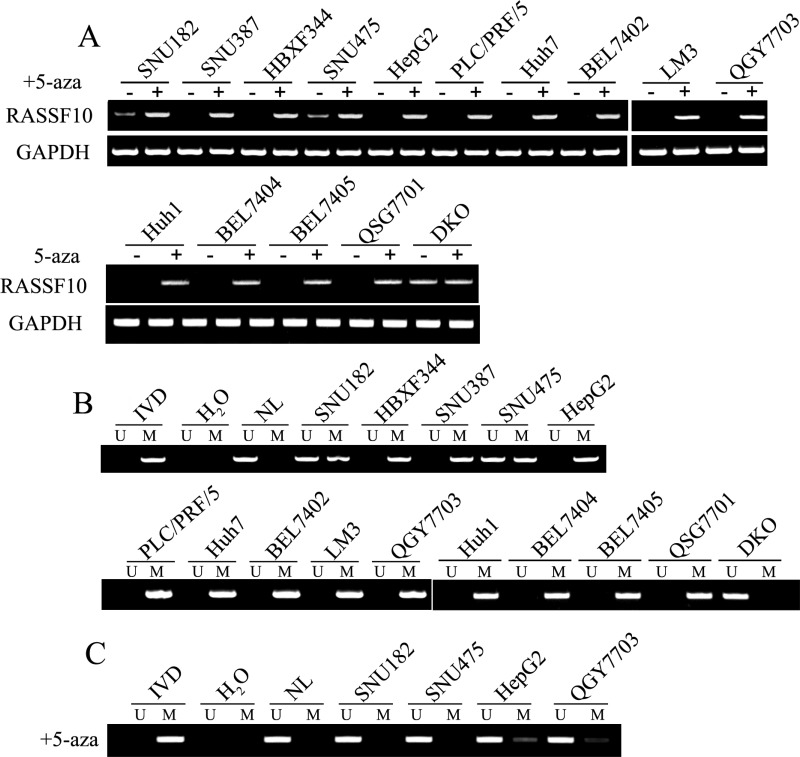
The expression and methylation status of RASSF10 in HCC cells (A) Expression of RASSF10 was detected by semi-quantitative RT-PCR. 5-aza: 5-aza-2′-deoxycytidine; GAPDH: The internal control of RT-PCR. SNU182, SNU387, HBXF344, SNU475, HepG2, BEL7402, LM3, Huh7, PLC/PRF5, QGY7703, Huh1, BEL7402, BEL7405 and QSG7701 are HCC cell lines, DKO is colorectal cancer cell line. (–): absence of 5-aza, ( + ): presence of 5-aza. (B) MSP results of RASSF10 in HCC cell lines and DKO cells. U: unmethylated alleles, M: methylated alleles; IVD: In vitro methylated DNA, methylation control; NL: normal peripheral lymphocytes DNA, unmethylation control.(C) MSP results of RASSF10 after 5-aza treatment in HCC cell lines.

**Figure 2:**
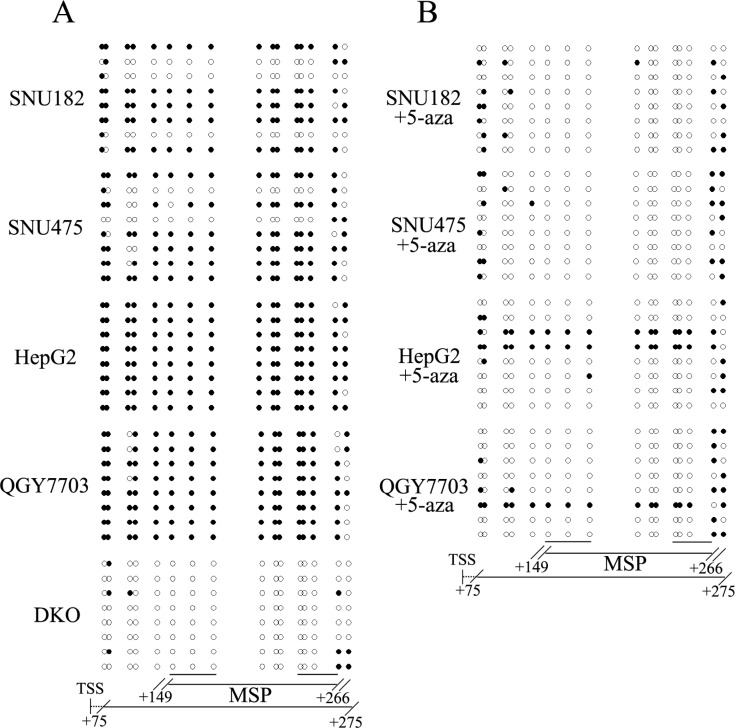
The bisulfite sequencing results of RASSF10 (A) Bisulfite sequencing results of RASSF10 in SNU182, SNU475, HepG2, QGY7703 and DKO cells. Double-headed arrow: MSP PCR product spanning 116 bp. Filled circle: methylated CpG site; open circle: unmethylated CpG site. TSS: transcriptional start site. (B) Bisulfite sequencing results of RASSF10 after 5-aza treatment in SNU182, SNU475, HepG2 and QGY7703 cells. Double-headed arrow: MSP PCR product spanning 116 bp. Filled circle: methylated CpG site; open circle: unmethylated CpG site. TSS: transcriptional start site.

The expression of RASSF10 was detected in HCC cell lines by semi-quantitative RT-PCR. As shown in Figure 1A, loss of RASSF10 expression was found in SNU387, HBXF344, HepG2, PLC/PRF/5, Huh7, BEL7402, LM3, QGY7703, SNU449, SMMC7721, BEL7404, BEL7405, Sk-hep1, QSG7701 and Huh1 cells and reduced expression was found in SNU182 and SNU475 cells. RASSF10 was highly expressed in DKO cells. Promoter region methylation was detected by methylation specific PCR (MSP). Complete methylation was found in SNU387, HBXF344, HepG2, PLC/PRF/5, Huh7, BEL7402, LM3, QGY7703, SNU449, SMMC7721, Sk-hep1, Huh1, BEL7404, BEL7405 and QSG7701 cells, partial methylation was found in SNU182 and SNU475 cells, and unmethylation in DKO cells (Figure 1B). These results demonstrated that loss or reduced expression of RASSF10 correlated with promoter region methylation. Treatment with the DNA methylation transferase inhibitor 5-aza-2′-deoxycytidine (5-aza) restored RASSF10 expression in SNU387, HBXF344, HepG2, PLC/PRF/5, Huh7, BEL7402, LM3, QGY7703 SNU449, SMMC7721, Sk-hep1, Huh1, BEL7404, BEL7405 and QSG7701 cells and increased RASSF10 expression in SNU182 and SNU475 cells (Figure 1A). The above results suggest that RASSF10 expression is regulated by promoter region methylation. To further validate the efficiency of the MSP primers and the methylation density in the promoter region, sodium bisulfite sequencing (BSSQ) was performed in SNU182, SHU475, HepG2, QGY7703 and DKO cells. The BSSQ results were consistent with the MSP results (Figure 2A). As shown in Figure 1C and 2B, the direct effect of 5-aza induction of RASSF10 expression was validated by MSP and BSSQ after 5-aza treatment in SNU182, SHU475, HepG2 and QGY7703 cells.

### RASSF10 is frequently methylated in human primary hepatocellular carcinoma

**Figure 3:**
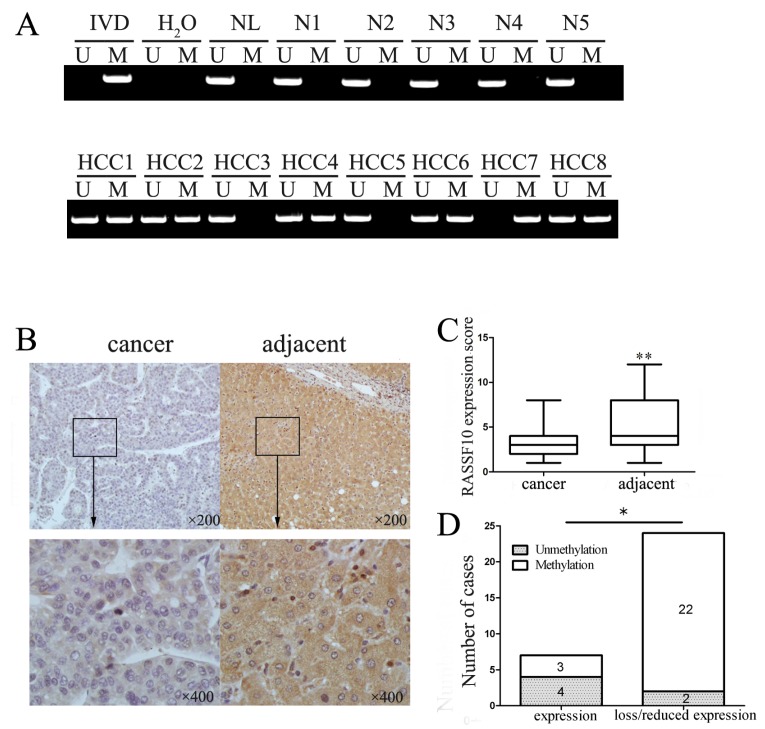
Representative results of RASSF10 methylation and expression in primary HCC (A) Representative MSP results of RASSF10 in normal human liver (N1-N5) and primary HCC (HCC1-HCC8) tissue samples. N: normal liver tissue samples. HCC: hepatocellular carcinoma tissue samples. (B) Representative IHC results of RASSF10 expression in HCC and adjacent tissue samples (upper boxes, 200×; lower boxes 400×). (C) RASSF10 expression scores are shown as box plots, horizontal lines represent the median score; the bottom and top of the boxes represent the 25th and 75th percentiles, respectively; vertical bars represent the range of data. Expression of RASSF10 was different between adjacent tissue samples and HCC tissue samples in 31 cases of primary HCC tissue samples. **P<0.01. (D) The expression of RASSF10 and DNA methylation status is shown as a bar diagram. Reduced expression of RASSF10 was significantly associated with promoter region hypermethylation. *P<0.05.

To determine the methylation status of RASSF10 in human primary HCC, 69 cases of primary HCC and 20 cases of normal liver tissue samples were examined by MSP. RASSF10 was methylated in 82.6% (57/69) of primary HCC samples, but no methylation was detected in normal liver tissue samples (Figure 3A). As shown in Table 1, methylation of RASSF10 was significantly associated with tumor size (P < 0.05) and tumor stage (P < 0.05), but no association was found between RASSF10 methylation and age, gender, HBV infection, cirrhosis, lymph node metastasis and cell differentiation. The expression of RASSF10 was evaluated by immunohistochemistry (IHC) in 31 cases of available matched primary HCC and adjacent tissue samples. Staining of RASSF10 was mainly localized in the cytoplasm and its expression was significantly reduced in primary HCC compared to adjacent tissue samples (p<0.05, Figure 3B and C). In 31 cases of available primary hepatic cancer samples, loss or reduced expression of RASSF10 was found in 24 cases. Of these 24 case samples, 22 cases were methylated and 2 cases were unmethylated. Loss or reduced expression of RASSF10 was significantly associated with promoter region hypermethylation (p<0.05, Figure 3D). These results indicate that RASSF10 expression is regulated by promoter region methylation in primary HCC.

**Table 1:** Clinic-pathological features and RASSF10 methylation in human primary HCC tissue samples

Clinical Factor	No. 69	RASSF10 methylation status		P value
		methylation n=57(82.6%)	unmethylation n=12(17.3%)	
**Age**				0.493
<50	23	18	5	
≥50	46	39	7	
**Gender**				0.492
F	10	50	9	
M	59	7	3	
**HBV**				0.569
Yes	38	30	8	
No	31	27	4	
**Cirrhosis**				0.945
Yes	31	26	5	
No	38	31	7	
**Lymph node metastasis**				0.607
Yes	6	4	2	
No	63	53	10	
**Differentiation**				0.413
High	5	5	0	
Moderate	54	43	11	
Low	10	9	1	
**Tumor size**				0.001*
≤5cm	26	17	9	
＞5cm	43	40	3	
**TNM Stage**				0.012*
I—II	22	14	8	
III—IV	47	43	4	

Chi-square test

* P<0.05 was regarded as a significant difference.

### Restoration of RASSF10 expression suppresses proliferation of HCC cells

**Figure 4:**
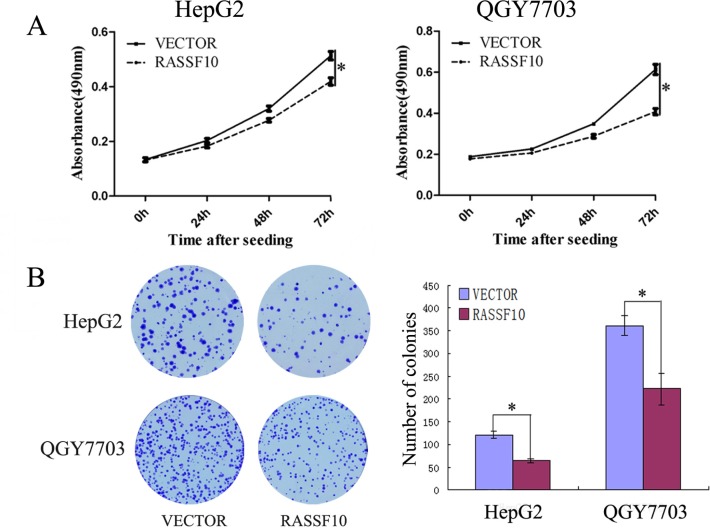
The effect of RASSF10 on HCC cell proliferation (A) Growth curves represent the effects of unexpressed and re-expressed RASSF10 in HepG2 and QGY7703 cells analyzed by the MTT assay. Each experiment was repeated in triplicate. * P < 0.05. (B) Colony formation results show that the colony number was reduced by re-expression of RASSF10 in HepG2 and QGY7703 cells. Each experiment was repeated in triplicate. The average number of tumor clones is represented by the bar diagram. * P < 0.05.

To evaluate the effects of RASSF10 on HCC progression, cell viability and colony formation assays were employed. As shown in Figure 4A, cell viability was determined by the MTT assay. The OD values were 0.498 ± 0.017 vs. 0.432 ± 0.021 (P<0.05) in HepG2 cells and 0.519 ± 0.028 vs. 0.320 ± 0.014 (P<0.05) in QGY703 cells before and after restoration of RASSF10 expression. The cell viability was significantly reduced. The colony numbers were 121 ± 8 vs. 64 ± 4 in HepG2 cells and 362 ± 22 vs. 222 ± 34 in QGY7703 cells before and after re-expression of RASSF10. The colony number was significantly reduced after re-expression of RASSF10 in HCC cells (all P<0.05, Figure 4B). These results demonstrate that RASSF10 suppresses the proliferation of HCC cells.

### RASSF10 induces G2/M phase arrest and sensitizes HCC cells to docetaxel

**Figure 5:**
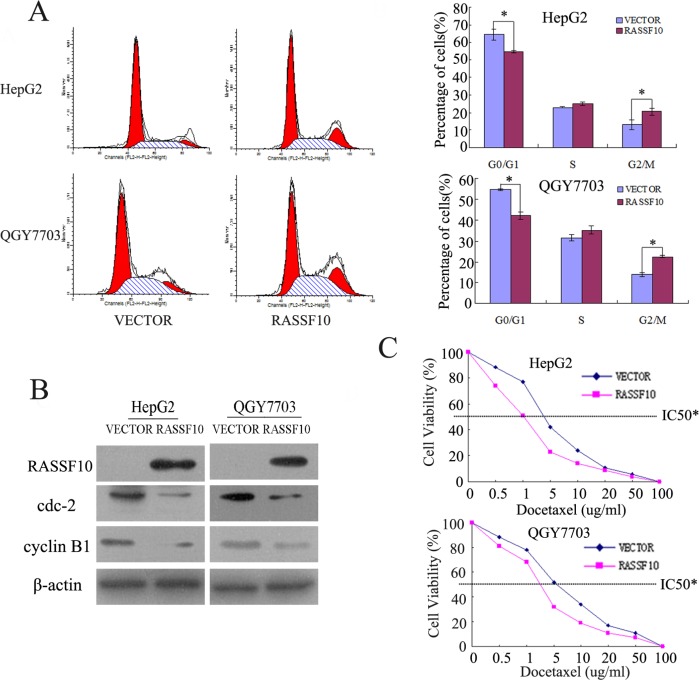
The effect of RASSF10 on cell cycle and the sensitivity of HCC cells to docetaxel (A) Cell phase distribution in RASSF10 unexpressed and re-expressed HepG2 and QGY7703 cells. The ratios are presented by bar diagram. Each experiment was repeated three times. * P < 0.05. (B) The expression of RASSF10, cyclin B1 and cdc-2 was detected by western blot in RASSF10 unexpressed and re-expressed HepG2 and QGY7703 cells. β-actin: internal control. (C) The cell viability analysis shows the sensitivity of RASSF10 re-expressed or unexpressed HepG2 and QGY7703 cells to docetaxel. *P <0.05.

The role of RASSF10 in the cell cycle was analyzed by flow cytometry. As shown in Figure 5A, the distribution of cell phase in RASSF10 unexpressed and re-expressed HepG2 cells was 64.22 ± 3.0% vs. 54.57 ± 0.7% in G0/G1 phase, 22.88 ± 0.3% vs. 24.96 ± 1.1% in S phase and 12.9 ± 2.7% vs. 20.48 ± 1.7% in G2/M phase. In QGY7703 cells, the cell phase distribution was 54.65 ± 0.4% vs. 42.26 ± 1.8% in G0/G1 phase, 31.54 ± 1.3% vs. 35.28 ± 2.1% in S phase and 13.81 ± 1.0% vs. 22.45 ± 0.5% in G2/M phase before and after restoration of RASSF10 expression. The G0/G1 phase was significantly reduced and the G2/M phase was significantly increased before and after re-expression of RASSF10 in HCC cells (all P<0.05). The G1/S checkpoint was not obviously affected by RASSF10, as S phase was not changed significantly (P>0.05). The effect of RASSF10 on the arrest of the G2/M checkpoint was further validated by detecting G2/M phase related proteins in HCC cells. The expression levels of cyclin B1 and cdc-2, important G2/M checkpoint regulators, were dramatically reduced after re-expression of RASSF10 in HepG2 and QGY7703 cells (Figure 5B).

Docetaxel, a microtubule inhibitor, exerts its effects on the G2/M checkpoint. To determine whether RASSF10 is involved in docetaxel sensitivity, we examined the cell viability of RASSF10 unexpressed and re-expressed HepG2 and QGY7703 cells after treatment with docetaxel. The IC50 of docetaxel was 2.759 ± 0.079 vs. 1.008 ± 0.066 μg/ml (P < 0.05) in HepG2 cells and 4.115 ± 0.172 vs. 2.044 ± 0.144 μg/ml (P < 0.05) in QGY7703 cells before and after re-expression of RASSF10 (Figure 5C). These results indicate that RASSF10 sensitizes HCC cells to docetaxel.

### RASSF10 induces cell apoptosis and suppresses HCC cell growth by activating P53 signaling

**Figure 6:**
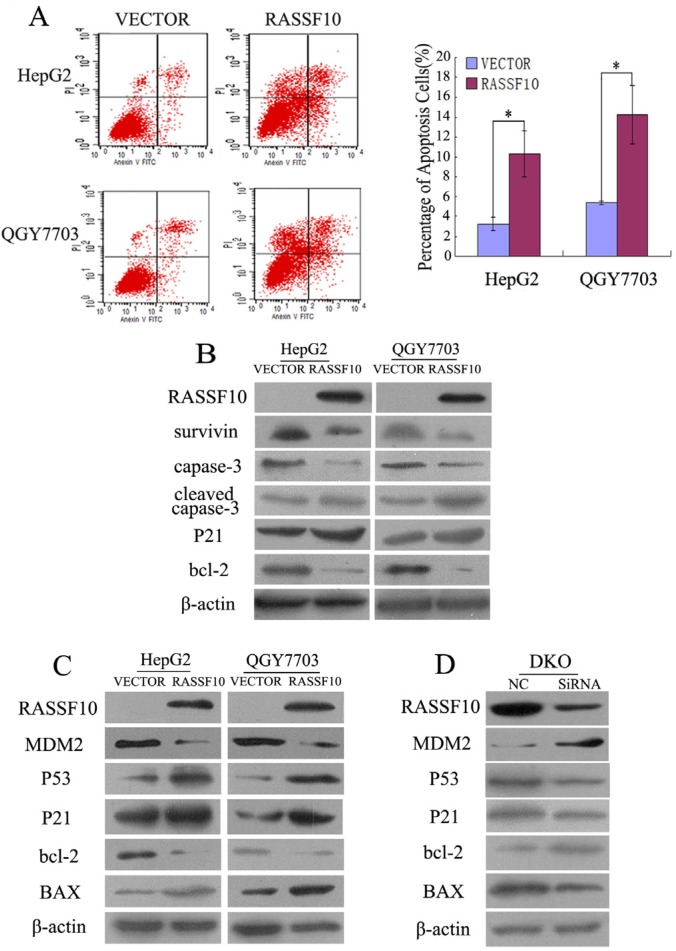
The role of RASSF10 in apoptosis and P53 signaling in human HCC cells (A) Flow cytometry results show the role of RASSF10 in apoptosis in HepG2 and QGY7703 cells. * P < 0.05. (B) Western blots show the effects of RASSF10 on survivin, capase-3, cleaved capase-3, P21 and bcl-2 expression in HepG2 and QGY7703 cells. β-actin: internal control. (C) Western blots demonstrate the role of RASSF10 in MDM2, P53, P21, bcl-2 and BAX expression in HepG2 and QGY7703 cells. β-actin: internal control. (D) The expression of MDM2, P53, P21, bcl-2 and BAX was detected by western blot after knocking down RASSF10 in DKO cells. β-actin: internal control.

To explore the role of RASSF10 in apoptosis, flow cytometry was performed. The percentage of apoptotic cells was 3.26 ± 0.66% vs 10.31 ± 2.38% in HepG2 cells (P<0.05) and 5.37 ± 0.16% vs 14.26 ± 3.00% in QGY7703 cells (P<0.05) before and after re-expression of RASSF10. These results suggest that RASSF10 induced cell apoptosis in HCC cells (Figure 6A). To further explore the mechanisms of RASSF10 in HCC cell apoptosis, survivin, capase-3, cleaved capase-3, P21 and bcl-2 levels were examined by western blot before and after re-expression of RASSF10. The expression levels of cleaved capase-3 and P21 were increased, while surviving, capase-3 and bcl-2 were reduced after re-expression of RASSF10 in HepG2 and QGY7703 cells (Figure 6B).

Our previous study reported that RASSF10 suppresses colorectal cancer growth by activating P53 signaling [13]. To understand the mechanism of RASSF10 in HCC, MDM2, P53, P21, bcl-2 and BAX, the key components of P53 signaling, were examined by western blot before and after re-expression of RASSF10 in HepG2 and QGY7703 cells. The expression levels of P53, P21 and BAX were increased, and MDM2 and bcl-2 were reduced after re-expression of RASSF10 (Figure 6C). These results indicate that P53 signaling is activated by RASSF10 in HCC cells. To further validate the growth inhibiting effect of RASS10 by activating p53 signaling, siRNA knockdown technique was applied in RASSF10 highly expressed DKO cells. As shown in Figure 6D, the expression of P53, P21 and BAX were reduced, MDM2 and bcl-2 were increased by knocking down RASSF10. The results further suggest that RASSF10 suppresses HCC cell growth by activating p53 signaling.

## DISCUSSION

RASSF10 has been reported to be frequently methylated in different malignancies and is regarded as a tumor suppressor [13, 22-24, 27]. However, the mechanism of RASSF10 in human HCC remains unclear. Our study found that RASSF10 was frequently methylated in human HCC and its expression was regulated by promoter region methylation. Methylation of RASSF10 was associated with tumor size and stage. Restoration of RASSF10 suppressed cell proliferation and induced apoptosis in human HCC. It has been reported that knockdown of RASSF10 increases mitosis in A549 lung cancer cells [26]. Our previous study also found that RASSF10 induces G2/M arrest in human colorectal cancer and esophageal cancer [13, 25]. The effects of RASSF10 on G2/M arrest were verified in human HCC in this study. The sensitivity of HepG2 and QGY7703 cells to docetaxel increased after re-expression of RASSF10. These results suggest that methylation of RASSF10 may serve as a docetaxel resistant marker in human HCC. Similar to our previous study in human colorectal cancer, RASSF10 suppressed human HCC proliferation by activating p53 signaling.

In conclusion, RASSF10 is frequently methylated in human HCC and the expression of RASSF10 is regulated by promoter region methylation. Methylation of RASSF10 is associated with tumor size and TNM stage, and it may serve as a docetaxel resistant marker in human HCC. RASSF10 suppresses human HCC growth by activating P53 signaling.

## MATERIAL AND METHODS

### Human tissue samples and cell lines

A total of 69 cases of primary hepatocellular carcinoma were surgically resected. Twenty cases of normal liver tissue samples were collected from noncancerous patients. All samples were stored at −80°C. Tumor stage was determined according to the American Joint Committee on Cancer (AJCC) TNM Staging for liver tumors (7th ed., 2010). Thirty-one cases of cancer tissue paraffin blocks were available with matched adjacent tissue samples. All samples were collected under the guidelines approved by the institutional review board of the Chinese PLA General Hospital. HCC cell lines SNU182, SNU387, HBXF344, SNU475, HepG2, PLC/PRF/5, Huh7, BEL7402, LM3, QGY7703, SNU449, SMMC7721, Sk-hep1, Huh1, BEL7404, BEL7405 and QSG7701 cel lines were previously established from human primary HCC. DKO is a DNMT1 and DNMT3b double knockout cell stemed from HCT116 cell, a colorectal cancer cell line. All cells were maintained in 90% RPMI 1640 (Invitrogen, Carlsbad, CA) supplemented with 10% fetal bovine serum.

### 5-aza-2′-deoxycytidine (5-aza) treatment

Cell lines were split to a low density (30% confluence) 12h before treatment. Cells were treated with 5-aza-2′-deoxycytidine (5-aza) (Sigma, St. Louis, MO, USA) at a concentration of 2 μM. Growth medium conditioned with 5-aza at 2μM was exchanged every 24 hours for a total of 96 hours of treatment.

### RNA isolation and semi-quantitative RT-PCR

Total RNA was isolated by Trizol reagent (Invitrogen, Carlsbad, USA). First strand cDNA was synthesized according to the manufacturer's instructions (Invitrogen, Carlsbad, CA). Semi-quantitative reverse transcription-PCR (RT-PCR) was performed as described previously [28]. RT-PCR primers are as follows: 5′- GTCGTCCTGTTCGTCCACTT-3′ (F), 5′- TGTCCTGCACGTAGTTGACC-3′ (R). RT-PCR was amplified for 34 cycles. GAPDH was amplified for 25 cycles and used as an internal control.

### DNA extraction, methylation-specific PCR (MSP) and bisulfite sequencing (BSSQ)

DNA was prepared by the proteinase K method. Bisulfite treatment was carried out as previously described [28]. Primers for MSP and BSSQ were designed around the transcription start site.

The sequences of the MSP primers are as follows: 5′-GTGTTATGGATTTTTTGGAAAAGAAGATATT-3′ (UF), 5′-TCCTCCAAAAACACTCACACAACATCA-3′ (UR), 5′-GTTATGGATTTTTCGGAAAAGAAGATATC-3′ (MF) and 5′-CTCCAAAAACACTCGCACAACGTCG-3′ (MR).

The sequences of the BSSQ primers are as follows: 5′-GAGTTATTGGGTTGTTTTTGTTG-3′ (F) and 5′-CRACAACCRTCCTCCAAAAAC-3′ (R).

### Immunohistochemistry

Immunohistochemistry (IHC) was performed in primary HCC and paired adjacent tissue samples. The dilution of RASSF10 antibody (Lifespan Bioscience, WA, USA) was 1:25. Rabbit polyclonal antibody against RASSF10 was used. IHC was performed and evaluated as described previously [9]. The staining intensity and extent of the staining area were graded according to the German semi-quantitative scoring system as described previously [29, 30].

### Expressional vector construction and transfection

The CDS region of RASSF10 was amplified by RT-PCR and cloned into the pcDNA3.1 expression vector. Primer sequences are as follows: 5′-GATCGAATTC GCCACCATGGATCCTTCGGAAAAGAAGATATC-3′ (F) and 5′-GATCTCTAGACTACACAAGGGATTCGCACATG-3′ (R). RASSF10 cDNA was then subcloned into the pLenti6-GFP lentivirus expression vector. RASSF10 expressing lentiviral or empty vectors were packaged using the ViraPowerTM lentiviral expression system (Invitrogen, San Diego, CA, USA). Lentivirus was added to the supernatant of HepG2 and QGY7703 cell culture medium, and RASSF10 stably expressed cells were selected by blasticidin (2 μg/ml, Invitrogen).

### Colony formation assay

Cells were seeded into 6-well culture plates at a density of 800 cells per well in triplicate and cultured for 2 weeks. Cells were then fixed with 75% ethanol for 30 minutes, stained with 0.2% crystal violet (Beyotime, Nanjing, China) for 20 minutes and counted.

### Cell viability assay

Cells were seeded into 96-well plates at 2×103 cells/well, and the cell viability was measured by the MTT assay at 0, 24, 48 and 72h (KeyGENBiotech, Nanjing, China). Absorbance was measured on a microplate reader (Thermo Multiskan MK3, MA, USA) at a wavelength of 490 nm.

The sensitivity of HCC cells to docetaxel was evaluated by detecting the IC50 of RASSF10 unexpressed and re-expressed gastric cancer cells treated with docetaxel at concentrations of 0, 0.5, 1, 5, 10, 20, 50 and 100 μg/ml for 48 hours. IC50 was defined as the concentration that was required for 50% inhibition of cell growth. The percentage of viable cells (%) = [A490 (treated)-A490 (blank)]/[A490 (control)-A490 (blank)] ×100%.

### Flow cytometry analysis

For cell cycle analysis, cells were fixed with 70% ethanol and stained with 50 mg/ml propidium iodide (KeyGEN Biotech, Jiangsu, China). The cells were then sorted by a FACS Caliber (BD Biosciences, San Jose, CA) and analyzed by the Modfit software (Verity Software House, ME, USA).

For apoptosis analysis, the Annexin V-FITC/PI Apoptosis Detection Kit (KeyGen Biotechnology, China) was used according to manufacturer's instructions. Each sample was analyzed by flow cytometry with a FACScan Flow Cytometer (Becton-Dickinson Biosciences, Mansfield, MA).

### Western blot

Western blot was performed as described previously [28]. Antibodies were diluted according to manufacturer's instructions. The primary antibodies used were as follows: RASSF10 (Lifespan Bioscience, WA, USA), MDM2 (Protein Tech Group, Chicago, IL, USA), P53 (Protein Tech Group, Chicago, IL, USA), capase-3 (Protein Tech Group, Chicago, IL, USA), cleaved caspase-3 (Protein Tech Group, Chicago, IL, USA), BAX (Protein Tech Group, Chicago, IL, USA), bcl-2 (Protein Tech Group, Chicago, IL, USA), cyclinB1 (Protein Tech Group, Chicago, IL, USA), cdc-2 (Protein Tech Group, Chicago, IL, USA), survivin (Protein Tech Group, Chicago, IL, USA), P21 (Abcam, MA, USA) and β-actin (Bioworld Tech, MN, USA).

### SiRNA knockdown assay

SiRNA knockdown assay was performed according to the manufacturer's instructions. The sequences of siRNA targeting RASSF10 and RNAi Negative Control Duplex are as follow: siRNA duplex (sense: 5′-GCGAAGAGCAAGAGAAUGUTT-3′; antisense: 5′-ACAUUCUCUUGCUCUUCGCTT-3′); RNAi negative control duplex (sense: 5′-UUCUCCGAACGUGU CACGUTT-3′; antisense: 5′-ACGUGACACGUUC GGAGAATT-3′) (Gene Pharma Co, Shanghai, China).

### Statistical analysis

SPSS 17.0 software was used for data analysis. All data are presented as means ± standard deviation (SD) of at least three independent experiments and analyzed using the student's t test. P<0.05 is regarded as a significant difference.

## References

[1] Ferlay J, Soerjomataram I, Dikshit R, Eser S, Mathers C, Rebelo M, Parkin DM, Forman D, Bray F (2015). Cancer incidence and mortality worldwide: Sources, methods and major patterns in globocan 2012. Int J Cancer.

[2] Fearon ER, Vogelstein B (1990). A genetic model for colorectal tumorigenesis. Cell.

[3] Vogelstein B, Papadopoulos N, Velculescu VE, Zhou S, Diaz LA, Kinzler KW (2013). Cancer genome landscapes. Science.

[4] Guo M, Ren J, Brock MV (2008). Promoter methylation of hin-1 in the progression to esophageal squamous cancer. Epigenetics.

[5] Guo M, Ren J, House MG, Qi Y, Brock MV, Herman JG (2006). Accumulation of promoter methylation suggests epigenetic progression in squamous cell carcinoma of the esophagus. Clin Cancer Res.

[6] Yang B, Guo M, Herman JG, Clark DP (2003). Aberrant promoter methylation profiles of tumor suppressor genes in hepatocellular carcinoma. Am J Pathol.

[7] Harder J, Opitz OG, Brabender J, Olschewski M, Blum HE (2008). Quantitative promoter methylation analysis of hepatocellular carcinoma, cirrhotic and normal liver. Int J Cancer.

[8] Revill K, Wang T, Lachenmayer A, Kojima K, Harrington A, Li J, Hoshida Y (2013). Genome-wide methylation analysis and epigenetic unmasking identify tumor suppressor genes in hepatocellular carcinoma. Gastroenterology.

[9] Jia Y, Yang Y, Liu S, Herman JG, Lu F, Guo M (2010). Sox17 antagonizes wnt/beta-catenin signaling pathway in hepatocellular carcinoma. Epigenetics.

[10] Zhu H, Wu K, Yan W, Hu L, Yuan J, Dong Y, Li Y, Jing K, Yang Y, Guo M (2013). Epigenetic silencing of dach1 induces loss of transforming growth factor-beta1 antiproliferative response in human hepatocellular carcinoma. Hepatology.

[11] Zhang X, Yang Y, Liu X, Herman JG, Brock MV, Licchesi JD, Yue W (2013). Epigenetic regulation of the wnt signaling inhibitor dact2 in human hepatocellular carcinoma. Epigenetics.

[12] Djos A, Martinsson T, Kogner P, Caren H (2012). The rassf gene family members rassf5, rassf6 and rassf7 show frequent DNA methylation in neuroblastoma. Mol Cancer.

[13] Guo J, Yang Y, Linghu E, Zhan Q, Brock MV, Herman JG, Zhang B, Guo M (2015). Rassf10 suppresses colorectal cancer growth by activating p53 signaling and sensitizes colorectal cancer cell to docetaxel. Oncotarget.

[14] Hill VK, Underhill-Day N, Krex D, Robel K, Sangan CB, Summersgill HR, Morris M, Gentle D, Chalmers AD (2011). Epigenetic inactivation of the rassf10 candidate tumor suppressor gene is a frequent and an early event in gliomagenesis. Oncogene.

[15] Kudo T, Ikeda M, Nishikawa M, Yang Z, Ohno K (2012). The rassf3 candidate tumor suppressor induces apoptosis and g1-s cell-cycle arrest via p53. Cancer Res.

[16] Schagdarsurengin U, Richter AM, Wohler C, Dammann RH (2009). Frequent epigenetic inactivation of rassf10 in thyroid cancer. Epigenetics.

[17] Katase N, Gunduz M, Beder L, Gunduz E, Lefeuvre M, Hatipoglu OF, Borkosky SS, Tamamura R, Tominaga S, Yamanaka N, Shimizu K (2008). Deletion at dickkopf (dkk)-3 locus (11p15.2) is related with lower lymph node metastasis and better prognosis in head and neck squamous cell carcinomas. Oncol Res.

[18] Ahn J, Yu K, Stolzenberg-Solomon R, Simon KC, McCullough ML, Gallicchio L, Jacobs EJ, Ascherio A, Helzlsouer K, Jacobs KB, Li Q, Weinstein SJ, Purdue M (2010). Genome-wide association study of circulating vitamin d levels. Hum Mol Genet.

[19] Bernardini M, Lee CH, Beheshti B, Prasad M, Albert M, Marrano P, Begley H, Shaw P, Covens A, Murphy J, Rosen B, Minkin S, Squire JA (2005). High-resolution mapping of genomic imbalance and identification of gene expression profiles associated with differential chemotherapy response in serous epithelial ovarian cancer. Neoplasia.

[20] Talwalkar VR, Scheiner M, Hedges LK (1998). Microsatellite instability in malignant melanoma. Cancer Genet Cytogenet.

[21] Chen J, Xu J, Ying K, Cao G, Hu G, Wang L, Luo C, Lou M, Mao Y (2004). Molecular cloning and characterization of a novel human btb domain-containing gene, btbd10, which is down-regulated in glioma. Gene.

[22] Dansranjavin T, Wagenlehner F, Gattenloehner S, Steger K, Weidner W (2012). Epigenetic down regulation of rassf10 and its possible clinical implication in prostate carcinoma. Prostate.

[23] Wei Z, Chen X, Chen J, Wang W, Xu X, Cai Q (2013). Rassf10 is epigenetically silenced and functions as a tumor suppressor in gastric cancer. Biochem Biophys Res Commun.

[24] Helmbold P, Richter AM, Walesch S, Skorokhod A, Marsch W (2012). Rassf10 promoter hypermethylation is frequent in malignant melanoma of the skin but uncommon in nevus cell nevi. J Invest Dermatol.

[25] Lu D, Ma J, Zhan Q, Li Y, Qin J, Guo M (2014). Epigenetic silencing of rassf10 promotes tumor growth in esophageal squamous cell carcinoma. Discov Med.

[26] Richter AM, Walesch SK, Wurl P (2012). The tumor suppressor rassf10 is upregulated upon contact inhibition and frequently epigenetically silenced in cancer. Oncogenesis.

[27] Hesson LB, Dunwell TL, Cooper WN, Catchpoole D, Brini AT, Chiaramonte R, Griffiths M, Chalmers AD, Maher ER, Latif F (2009). The novel rassf6 and rassf10 candidate tumour suppressor genes are frequently epigenetically inactivated in childhood leukaemias. Mol Cancer.

[28] Yan W, Wu K, Herman JG, Brock MV, Fuks F, Yang L, Zhu H, Li Y, Yang Y, Guo M (2013). Epigenetic regulation of dach1, a novel wnt signaling component in colorectal cancer. Epigenetics.

[29] Cregger M (2006). Immunohistochemistry and quantitative analysis of protein expression. Arch Pathol Lab Med.

[30] Koo CL, Kok LF, Lee MY, Wu TS, Cheng YW, Hsu JD, Ruan A (2009). Scoring mechanisms of p16ink4a immunohistochemistry based on either independent nucleic stain or mixed cytoplasmic with nucleic expression can significantly signal to distinguish between endocervical and endometrial adenocarcinomas in a tissue microarray study. J Transl Med.

